# The impact of environmental factors on quality of life and symptoms of children with allergic rhinitis

**DOI:** 10.5935/1808-8694.20130102

**Published:** 2015-10-08

**Authors:** Therezita Maria Peixoto Patury Galvão Castro, Diogo Ramalho Tavares Marinho, Camila Carvalho Cavalcante

**Affiliations:** a Doctoral Student, Otorhinolaryngology Course, FCMSCSP (Assistant Professor, Otorhinolaryngology Course, UNCISAL, UFAL).; b Sixth-year medical student, UFAL (Sixth-year medical student, UFAL). Federal University of Alagoas - UFAL.

**Keywords:** assisted living facilities, perennial allergic rhinitis, quality of life

## Abstract

Allergic rhinitis is an inflammation of the nasal mucosa caused by exposure to allergens, which impairs the cognitive capabilities of the affected.

**Objective:**

To correlate the mean scores of quality of life of children and adolescents with symptoms of allergic rhinitis and the presence of household environmental factors described in the literature as allergy triggers.

**Method:**

This cross-sectional retrospective cohort study included 120 children and adolescents presenting clinical manifestations of allergic rhinitis. The subjects were divided into two groups based on the number of allergy-triggering environmental factors seen in their households. Scale PedsQL 4.0 was used to quantify quality of life and allow further comparisons between groups.

**Results:**

No statistically significant differences (*p* > 0.05) were seen in the PedsQL mean scores when participant quality of life was analyzed vis-a-vis triggering environmental factors. However, the incidence of allergy manifestations was higher in children exposed to more environmental factors.

**Conclusion:**

The studied environmental factors were not correlated with patient quality of life. However, the analysis of patient households and symptoms indicates the environment played a role in the onset of allergy events.

## INTRODUCTION

Allergic rhinitis is an inflammation of the nasal mucosa caused by exposure to allergens which, after sensitization, trigger an inflammatory response mediated by immunoglobulin E (IgE) that may result in chronic or recurrent symptoms^1^, ^2^. Prevalence has increased in the last decades partly due to greater environmental exposure, life style changes (longer permanence in enclosed spaces), and socioeconomic factors^3^, ^4^. Global statistics indicate prevalences of 30% to 40% in children and adolescents. In Brazil, studies have reported prevalences of 33% and 34% in schoolers aged six to seven and 13 to 14 years respectively^5^. Yet, allergic rhinitis (AR) is estimated to be underdiagnosed^6^.

The onset of allergic rhinitis clinical manifestations takes place more commonly during childhood, although cases of later onset have been reported by up to 30% of the patients^7^. The main symptoms include aqueous rhinorrhea, nasal obstruction/pruritus, sneezing and ocular symptoms such as pruritus and conjunctival hyperemia, which tend to resolve spontaneously or after treatment^1^.

Allergic sensitization has been correlated with the interaction of genetic and environmental factors, as well as weather conditions and local cultural aspects. Therefore, knowledge of regional profiles is relevant for the development of environmental control measures^8^.

Thorough clinical history is still the most valuable tool in the diagnosis of allergic rhinitis^9^. In environmental history, it is important to look for the conditions of the environment the patient lives in (including the subject's household and neighborhood, workplace, trips to day care centers and schools) in terms of the following aspects: age of household and school building, ventilation, type of flooring, presence of carpeting or rugs, draperies, shelves, materials and upholstery used in mattresses, pillows and blankets, presence of pets with fur, cockroaches, second-hand smoking, exposure to non-specific irritants, air-conditioning and maintenance of air-conditioning equipment, plants in the household, vegetation in external areas, and external pollution^10^.

Contact with allergens has been associated with inflammation in allergic diseases, mainly asthma and allergic rhinitis, and environmental control is of paramount importance in the treatment of these diseases, as recommended in national and international consensus papers^11^.

The main control measures include: avoid pillows and mattresses filled with silk floss or feathers; preferably well ventilated and sunny bedrooms; avoid carpets, rugs, draperies, and cushions; eradicate mold and moisture; avoid using brooms, dusters, and ordinary vacuum cleaners; avoid second-hand smoking; clothes and blankets must be washed and dried in the sun before used; avoid pets with fur or feathers^10^.

Recent studies described the negative impacts of AR upon learning, cognitive skills, memory, and psychosocial relations^10^. Behavior disorders such as restlessness, irritability, attention deficit, and diurnal sleepiness have also been reported in the literature. These symptoms may impair the child's ability to focus, and thus negatively affect performance at school^12^.

Another aspect to consider is the chronic nature of the disease, which affects the long-term quality of life of affected individuals and significantly increases the cost of medical care^2^.

Assessment of quality of life is a complex topic. Perceptions of quality of life change dynamically and may vary between individuals^13^. However, quality-of-life assessment may provide a better understanding of one's actual needs, as it measures the impact of disease and treatment from the standpoint of the patient^14^. Scales and instruments have been developed and used with this purpose in a wide array of circumstances^15^.

Health-related quality of life (HRQOL) scales must include multiple domains and assess, at least, the patients' physical, psychological (including cognitive and emotional aspects), and social dimensions, as recommended by the World Health Organization (WHO)^16^.

The Pediatric Quality of Life Inventory^TM^ (Peds-QL^TM^) instrument was idealized to verify the domains pertaining to physical, mental, and social health as per the propositions of the WHO, taking into consideration the role of the school and integrating the merits of generic and disease-specific approaches^17^.

The PedsQL version 4.0 scale includes a self-assessment tool for children and adolescents aged between five and 18 years and scales for parents of children and adolescents aged between two and 18 years^17^. The scales are divided into age ranges - 2-4, 5-7, 8-12 and 13-18 years. The items in the scales for each group are similar, and are tailored to consider the different levels of development of children and adolescents^18^, ^19^. Each scale comprises 23 items divided into four subscales: physical (eight items); emotional (five items); social (five items); and school (five items). Subject responses are graded between 0 and 4 (0 = never a problem; 1 = almost never a problem; 2 = sometimes a problem; 3 = often a problem; 4 = almost always a problem) and refer to how much of a problem each item had been during the past month^20^.

This study aims to correlate the mean quality of life scores in each domain of the PedsQL 4.0 attained by children and adolescents with clinical manifestations of allergic rhinitis and the presence of household environmental factors reported in the literature as triggers of allergy events and, secondarily, to assess whether these factors impact the intensity of symptoms experienced by the subjects.

## METHOD

This cross-sectional historical cohort study was approved by the Research Ethics Committee of the institution and given permit 005729/2011-84. The study was carried out from August 1, 2011 to July 31, 2012.

The 120 children and adolescents included in the study met the following enrollment criteria: age between five and 18 years; clinical manifestations of allergic rhinitis; treatment naive; no other comorbidities; residents of the community where the study was carried out. Subjects who refused to join the study and whose parents did not sign the informed consent term were excluded.

Bills were posted in the local health care service and community health agents informed the population about the study. Participants were explained what the study entailed and were presented the informed consent terms. A semistructured questionnaire prepared specifically for this study was answered by participants to assess the presence of clinical manifestations of allergic rhinitis and capture epidemiological information to ensure they met the enrollment criteria.

Participants were asked to answer a second, equally study-specific questionnaire based on ‘File A' from the Basic Health Care Information System of the Brazilian Ministry of Health^21^. This questionnaire also collected the environmental epidemiological information recommended by Ibiapina et al.^1^, Dykewicz & Hamilos^2^, and Silva et al.^5^ with the purpose of assessing the environment of the participants' households.

Quality of life was assessed through the Pediatric Quality of Life Inventory *-* PedsQL 4.0 (children and adolescent self-assessment scale) validated for the Brazilian population^18^. Depending on the level of education of the subjects, the self-assessment scales were read to each individual by trained interviewers. The Likert five-point scale (0 = never a problem; 1 = almost never a problem; 2 = sometimes a problem; 3 = often a problem; 4 = almost always a problem) was used with PedsQL 4.0. Responses were counted, reversed and converted into a scale from 0 to 100 (0 = 100; 1 = 75; 2 = 50; 3 = 25; 4 = 0). The summation of responses was then divided by the number of items. Higher scores indicated better quality of life^17^.

Overall quality of life was determined by the mean score in all domains, while psychosocial performance was assessed by the mean score calculated from the scores in the social, emotional, and school subscales^22^. Cronbach's alpha was used to estimate the internal consistency of the scale. Scales with reliability ≥ 0.70 are recommended to compare groups of patients, while scales with reliability of 0.90 are recommended to analyze the scores of individual patients^23^, ^24^.

Nine environmental factors have been reported in the literature as possible triggers of allergy episodes^20^: absence of sunlight in the room; carpeting or rugs; draperies; pets; on-bed plush toys; mosquito nets; house type; disposal of waste; house cleaning procedure. The median was calculated for these factors and patients were split into two groups based on the number of environmental factors present in each household.

The data was treated and analyzed using Microsoft Excel^®^ and EpiInfo^®^ version 3.5.1. Continuous variables were analyzed through Student's t-test, the Kruskal-Wallis test (non-parametric test); categorical variables were analyzed through the chi-square test or Fisher's exact test (non-parametric test). A confidence interval of 95% was considered and significance level was attributed for *p* < 0.05.

## RESULTS

Forty-eight (40%) subjects were males and 72 (60%) were females. None of the patients were active smokers, but 42 (35%) were second-hank smokers and lived with at least one active smoker.

**Graph 1:**
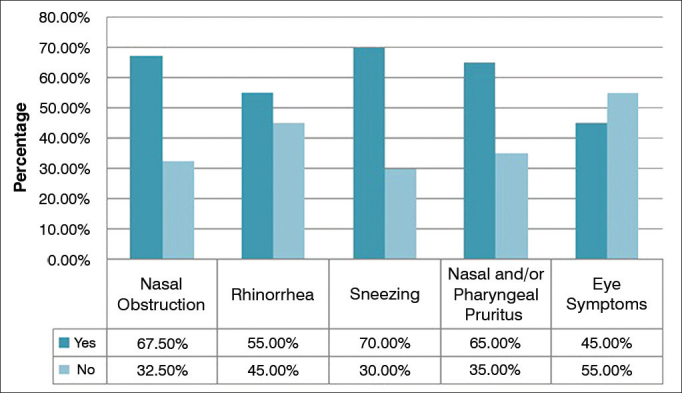
Distribution of patient clinical manifestations.

The most frequently reported clinical manifestation of allergic rhinitis was sneezing, seen in 84 (70%) patients, followed by nasal obstruction (67.50%), nasal and/or pharyngeal pruritus (65%), rhinorrhea (55%), and eye symptoms (45%) (Graph 1).

**Graph 2:**
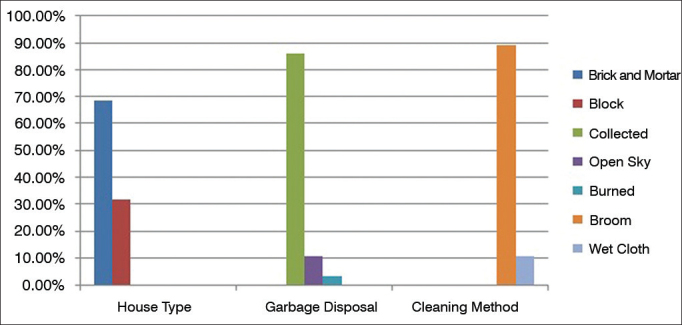
Patient household environmental conditions.

**Graph 3:**
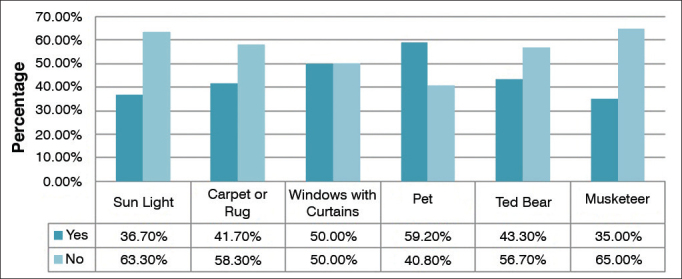
Patient bedroom environmental conditions.

In environmental analysis, the most frequently found triggering factor was the use of brooms, reported by 107 (89.2%) subjects (Graph 2). In bedrooms, the absence of sunlight was the most frequent factor, seen in 63.30% of the patient households, followed by pets (59.20%), windows with curtains (50.0%), plush pets on the bed (43.30%), carpeting or rugs (41.70%), and mosquito netting (35.0%) (Graph 3).

Table 1 shows the internal consistency of the PedsQL 4.0 scores for children and adolescents. The Cronbach's alpha for the physical, social, and school functioning subscales were above 0.70. Only the emotional subscale had an alpha under 0.7. Cronbach's alpha for the scale's total score was 0.9, which indicated excellent reliability.

**Table 1:** Internal consistency (Cronbach's alpha) of the Pediatric Quality of Life Inventory^TM^ 4.0 scale for children.

Item	Cronbach's alpha	N
Scale total score	0.9	120
Physical	0.774	120
Emotional	0.68	120
Social	0.712	120
School	0.769	120
Psychosocial	0.79	120

Table 2 shows the median value of triggering environmental factors of two groups of patients based on the number of environmental factors present in their households. More than half the patients (59.2%) had fewer than five environmental factors and 49 (40.8%) had five or more environmental factors (Table 3).

**Table 2:** Number of triggering environmental factors found in the patients' households.

Number of triggering environmental factors	Number of households	Percentage
1	2	1.7%
2	9	7.5%
3	33	27.5%
4*	27	22.5%
5	21	17.5%
6	14	11.7%
7	6	5.0%
8	7	5.8%
9	1	0.8%
Total	120	100%

* Median: 4.

**Table 3:** Distribution of patients according to the number of triggering environmental factors.

Patient Group	Number of subjects	Percentage	95% Confidence Interval
Fewer than 5 factors	71	59.2%	(49.8-68.0)%
5 or more factors	49	40.8%	(32.0-50.2)%
Total	120	100.0%	

Table 4 shows the mean scores in the PedsQL 4.0 subscales and correlates the quality of life of children and adolescents with clinical manifestations of allergic rhinitis to the number of environmental factors found in their households. No statistically significant differences were found between the groups of subjects in any of the scale domains (*p* > 0.05). The overall mean quality of life score was below 50 in both groups; when each subscale was analyzed separately, only social functioning had a score greater than 50.

**Table 4:** Mean scores in quality of life subscales for children and adolescents with clinical manifestations of allergic rhinitis.

Quality of Life Subscalesw	Group with fewer than five environmental factors		Group with more than five environmental factors		*p*
	Number of patients	Mean	Number of patients	Mean	
Physical	71	43.3539	49	41.0077	0.4193*
Emotional	71	44.2958	49	44.2857	0.8932*
Social	71	54.0141	49	52.1429	0.3937*
School	71	45.2817	49	40.9184	0.0881*
Psychosocial	71	47.8638	49	45.7823	0.4555*
Overall QoL	71	46.2952	49	44.1216	0.1530*

* *p* > 0.05; non-statistically significant difference. Kruskal-Wallis test applied to two groups.

In regards to clinical manifestations, five symptoms were considered: nasal obstruction, rhinorrhea, nasal and/or pharyngeal pruritus, ocular symptoms, and sneezing. The median number of symptoms was calculated (Table 5) and patients were split into two groups based on the number of clinical manifestations they experienced. Eighty-three (69.2%) patients had fewer than four symptoms and 37 (30.8%) had four or more symptoms (Table 6).

**Table 5:** Number of symptoms presented by the patients.

Number of symptoms	Number of patients	Percentage
1	5	4.2%
2	39	32.5%
3*	39	32.5%
4	22	18.3%
5	15	12.5%
Total	120	100%

* Median: 3.

**Table 6:** Distribution of patients based on the number of symptoms.

Symptoms	Number of subjects	Percentage	95% Confidence Interval
Patients with fewer than four symptoms	83	69.2%	(60.1-77.3)%
Patients with four or more symptoms	37	30.8%	(22.7-39.9)%
Total	120	100.0%	

Table 7 shows the number of patients and percentages in relation to the total population and correlates the number of manifested symptoms with the number of environmental factors present in their households. A significant share (84.5%) of the patients with fewer than five environmental factors had fewer than four symptoms. More than half (53.1%) of the patients with five or more environmental factors had four or more symptoms. Odds ratio calculations showed that subjects exposed to more than five environmental factors were 6.16 times more likely to have more than four symptoms than the group with fewer than five environmental factors 95% CI (2.6-14.4).

**Table 7:** Correlation between the number of environmental factors and number of symptoms presented by the patients.

Triggering environmental factors	Symptoms					
	Fewer than four		Four or more		Total	
	N	%	N	%	N	%
< 5	60	84.5	11	15.5	71	100
5 or more	23	46.9	26	53.1	49	100
Total	83	69.2	37	30.8	120	100

## DISCUSSION

The results have shown that the household environments included in the study did not comply with the health and sanitation requirements published in the literature for patients with allergic rhinitis^3^, ^4^, a finding compatible with most peripheral communities in Brazil. The clinical symptoms and the immune and allergic responses observed in the patients were consistent with the environmental findings.

It should be noted that most of the environmental factors described in the study can be mitigated by life style changes, such as no longer keeping plush toys in the bedroom or cleaning the house with a wet rag instead of a broom. Therefore, the implementation of awareness building programs may help design a healthier environment for allergic rhinitis patients.

No statistically significant differences (*p* > 0.05) were seen in the mean scores of the PedsQL 4.0 (version for the self-assessment of children and adolescents) subscales, when the quality of life of the participants was compared to the presence of the environmental factors assessed in the study. However, the overall mean quality of life score was below 50 for both groups; additionally, when each subscale was assessed separately, only social functioning had a score above 50, showing these patients were underperforming in all domains covered in the scale. The responses given to the items in the scale revealed these individuals were faced with significant difficulties having access to health care and education, in addition to having few leisure options and little available income. Additionally, both groups were made of non-healthy patients. Thus, the household environment alone could not explain the impacts upon the quality of life of the participants.

A multidisciplinary team is required to properly address the physical and psychosocial development of these children and adolescents, while the public authorities need to improve the infrastructure available in the communities where these patients live.

When the number of symptoms was assessed alongside the environmental factors identified in patient households, it was clear that patients residing in households with more environmental factors had more symptoms. Therefore, although the studied environmental factors did not introduce significant repercussions upon the quality of life of the patients, it may be said that they triggered symptoms, and should be included in the treatment strategy to control allergy events.

## CONCLUSION

The data indicated that the household environment of the patients included in this study was conducive to the establishment of the baseline disease in almost all cases. The number of improper health and sanitation standards was correlated with the number of symptoms presented by the patients. Thus, awareness building initiatives should be promoted to educate people on the importance of health and sanitation measures to reduce the exposure of patients to allergy triggering factors in their households.

No statistically significant differences were seen in the mean scores of the PedsQL 4.0 subscales when participant quality of life was compared to the presence of the environmental factors considered in this study.
